# Current state of stimulated Brillouin scattering microscopy for the life sciences

**DOI:** 10.1088/2515-7647/ad5506

**Published:** 2024-06-26

**Authors:** Alberto Bilenca, Robert Prevedel, Giuliano Scarcelli

**Affiliations:** 1 Biomedical Engineering Department, Ben-Gurion University of the Negev, 1 Ben Gurion Blvd, Be’er-Sheva 84105, Israel; 2 Ilse Katz Institute for Nanoscale Science and Technology, Ben-Gurion University of the Negev, 1 Ben Gurion Blvd, Be’er-Sheva 84105, Israel; 3 Cell Biology and Biophysics Unit, European Molecular Biology Laboratory, Heidelberg, Germany; 4 Fischell Department of Bioengineering, University of Maryland, College Park, MD 20742, United States of America

**Keywords:** optical microscopy, stimulated Brillouin scattering, biomechanical imaging

## Abstract

Stimulated Brillouin scattering (SBS) microscopy is a nonlinear all-optical imaging method that provides mechanical contrast based on the interaction of laser radiation and acoustical vibrational modes. Featuring high mechanical specificity and sensitivity, three-dimensional sectioning, and practical imaging times, SBS microscopy with (quasi) continuous wave excitation is rapidly advancing as a promising imaging tool for label-free visualization of viscoelastic information of materials and living biological systems. In this article, we introduce the theory of SBS microscopy and review the current state-of-the-art as well as recent innovations, including different approaches to system designs and data analysis. In particular, various performance parameters of SBS microscopy and its applications in the life sciences are described and discussed. Future perspectives for SBS microscopy are also presented.

## 1. Introduction

Stimulated Brillouin scattering (SBS) is a nonlinear interaction between light and propagating acoustic waves that arises primarily from electrostrictive and photoelastic processes [1–3]. In brief, incident light fields exert force on a material which in turn induces a strain field (via electrostriction) that can coherently drive an acoustic wave through the medium under energy and momentum conservation conditions; this leads to traveling gratings of dielectric permittivity in the medium (via photoelasticity) from which Doppler-shifted, SBS light can be detected. Based on SBS, stimulated Brillouin gain (SBG) and impulsive SBS (iSBS) spectroscopies were previously developed in the eighties and the nineties, enabling high spectral resolution measurements of the Brillouin spectrum of transparent materials [4–9 and references therein]. From such measurements, one can retrieve the material’s Brillouin shift and linewidth, which are intimately related to the material’s sound speed and attenuation, respectively. Provided that the refractive index and mass density of the sample are known, the longitudinal elastic and viscous moduli can also be evaluated. SBG spectroscopy has recently been extended to allow high spectral resolution measurements in tissue phantoms [10–12] as well as spectrotemporally optimized measurements in liquids [13]. By combining SBG (iSBS) spectroscopy and microscopy, (i)SBS microscopy has been devised, offering spectroscopic imaging of materials with high spectrometer resolution, but at limited spatiotemporal resolution [14–16]. Recent technological advances have enabled SBS imaging of the longitudinal elastic and viscous moduli of living systems with high mechanical sensitivity and specificity at down to a few tens of millisecond pixel dwell times, facilitating contactless, label-free, all-optical biomechanical visualization [17]. In this article, we therefore focus on SBS microscopy with (quasi) continuous wave (cw) excitation for biomechanical imaging. Spontaneous Brillouin scattering, which relies on thermally excited rather than stimulated acoustics phonons in the material, also enables biomechanical imaging through the use of Fabry–Perot interferometers or virtually imaged phased arrays (VIPAs), although it often trades off between spectral acquisition time, spectrometer resolution, optical resolution, and depth of field [18–33]. We note that in contrast to Raman scattering that probes optical phonons in the (sub)THz range, Brillouin scattering measures light scattering by MHz to GHz acoustic phonons and can also provide data to determine phonon dispersion.

Two variations of biomechanical SBS microscopy, which share a similar experimental arrangement, have been developed, cw and pulsed SBS microscopy [17, 34, 35]. cw-SBS microscopy involves the use of counter-propagating, pump and Stokes, single frequency, cw lasers. The laser beams are focused into a joint spot in the sample and their frequency difference is scanned around the acoustic vibrational frequency of materials in the samples (GHz range in biological specimens). The increase in the intensity of the Stokes beam (the SBG signal) owing to SBS is measured in each spectral bin using high sensitivity detection methods. By raster scanning the sample through the joint focus of the pump and Stokes beams and recording the SBG spectrum at each pixel, spectroscopic imaging is obtained. Analysis of the spatiospectral data is finally executed for obtaining mechanically related maps of the sample. As cw-SBS microscopy employs high average irradiation, caution has to be taken when living systems are imaged due to possible light absorption damage to the samples. A way to minimize linear photodamage is to reduce the incident average power on the sample by exciting SBS with long pump and Stokes pulses that overlap spatiotemporally in the sample. An important advantage of pulse excitation is the potential improvement of the performance of SBS microscopy in terms of imaging speed as it allows large SBG with sufficiently high (narrow) excitation linewidth.

SBS microscopy for biomechanical imaging is undergoing increasing research and developments as it offers (i) high specificity resulting from the high spectral resolution principally determined by the width of the Brillouin resonance of the materials, (ii) high imaging contrast owing to the significant suppression of the Rayleigh background signal in SBS and the high detection sensitivity, (iii) extended imaging depth and reduced photodamage to biological samples owing to the use of near infrared laser sources, (iv) practical biocompatible imaging times, with the potential of video rate imaging using long pulse and/or selective excitation of acoustic phonons, and (v) intrinsic optical sectioning.

This paper is organized as follows. We first introduce the theory of SBS microscopy, including the governing equations for SBG. Based on this we investigate the dependence of signal/noise on pixel dwell time and overall average excitation power, and the theoretical precision of the obtainable Brillouin shift and linewidth, and the mass density measurements. Furthermore, we discuss spectral effects induced by the numerical aperture (NA) of the objective lenses used. Next, experimental designs for cw-SBS and pulsed-SBS microscopy are described, discussing experimental considerations such as spectral and temporal resolution, signal/noise, and excitation energy. Then, SBS measurements and analysis methods in living systems are presented. Comparison with spontaneous Brillouin scattering and iSBS microscopy approaches is also discussed. Finally, conclusions and future perspectives for SBS microscopy are provided.

## 2. Theory of SBS microscopy

In this section, we focus on SBS in the linear, small-signal amplification regime for quasi-continuous-wave, counter-propagating pump and Stokes waves with parallel polarizations in an optically isotropic and lossless medium. This implies the temporal steady-state condition and the undepleted pump approximation, which are justified for long pulses with respect to the phonon lifetime (<∼1 ns at 780 nm) and given the low SBG in biological microscopy applications (∼1 × 10^−5^). In this backward scattering geometry, the longitudinal, or pressure, acoustic wave propagating in the medium corresponds to compressions and dilatations of the material along the propagation direction of the pump wave.

### 2.1. Governing equations

The propagation of the pump and Stokes waves in the medium is described by the electromagnetic wave equation

\begin{equation*}{\nabla ^2}\bar E - \frac{1}{{{c^2}}}\frac{{{\partial ^2}\bar E}}{{\partial {t^2}}} = \frac{1}{{{\varepsilon _0}{c^2}}}\frac{{{\partial ^2}\bar P}}{{\partial {t^2}}},\end{equation*}

where $\bar E$ is the electric field, *c* the speed of light, and $\varepsilon $_0_ the vacuum permittivity. $\bar P$ is the material polarization and reads as [1–3]

\begin{equation*}\bar P = {\bar P_{\text{L}}} + {\bar P_{{\text{NL}}}} = {\varepsilon _0}\chi \bar E + {\varepsilon _0}\frac{{{\gamma _{\text{e}}}}}{{{\rho _0}}}\Delta \rho \bar E.\end{equation*}

${\bar P_{\text{L}}}$ is the linear polarization owing to the material permittivity (or susceptibility $\chi $) and ${\bar P_{{\text{NL}}}}$ is the nonlinear polarization because of electrostriction, with $\gamma $_e_ being the electrostrictive constant, $\rho $_0_ the mean density of the medium, and $\Delta \rho $ the change in the density of the medium due to electrostriction (which is proportional to $\left\langle {\bar E \cdot \bar E} \right\rangle $, where the angular brackets denote a time average over an optical period). By substituting equation (2) into the electromagnetic wave equation equation (1), we get

\begin{equation*}{\nabla ^2}\bar E - \frac{{{n^2}}}{{{c^2}}}\frac{{{\partial ^2}\bar E}}{{\partial {t^2}}} = \frac{{{\gamma _{\text{e}}}}}{{{c^2}{\rho _0}}}\Delta \rho \bar E,\end{equation*}

with *n* denoting the refractive index of the material. The evolution of $\Delta \rho $ is connected with the propagation of the electrostrictive pressure is a dissipative medium, satisfying the viscous acoustic wave given by [1–3]

\begin{equation*}\frac{{{\partial ^2}\Delta \rho }}{{\partial {t^2}}} - \Gamma ^\prime{\nabla ^2}\frac{{\partial \Delta \rho }}{{\partial t}} - {v^2}{\nabla ^2}\Delta \rho = \nabla \cdot \bar f,\end{equation*}

where $\Gamma ^{\prime}$ is a damping parameter related to the kinematic viscosity, *v* is the speed of sound, and $\bar f = -0.5{\varepsilon _0}{\gamma _{\text{e}}}\nabla \langle \bar E \cdot \bar E \rangle$ is the ponderomotive force per unit volume arising in a dielectric placed in $\bar E$.

Equations (3) and (4) constitute a set of coupled equations that describes the interaction of the optical and acoustic waves and can be used to calculate the light intensity observed in SBS. Under the assumptions outlined in the beginning of this section and for plane pump, Stokes, and acoustic waves with envelopes *A_1_(z), A_2_(z)*, $\Delta \rho \left( z \right)$, angular frequencies ${\omega _1},{\omega _2},\Omega {\text{ }}$, and wave vectors **k**_1_, **k**_2_, **q**, this set of coupled equations is readily expressed as follows for calculating SBG

\begin{align*} &amp;\frac{{{\text{d}}{A_1}}}{{{\text{d}}z}} = 0 \nonumber\\ &amp;\frac{{{\text{d}}{A_2}}}{{{\text{d}}z}} = - i\kappa \Delta {\rho ^*}{A_1} \nonumber\\ &amp;\frac{{{\text{d}}\Delta \rho }}{{{\text{d}}z}} + \left( {\delta + i\beta } \right)\Delta \rho = i\eta {A_1}A_2^*.\end{align*}

Here, $i = \sqrt { - 1} $, $\kappa = {\omega _2}{\gamma _{\text{e}}}/2nc{\rho _0}$, $\eta = {\varepsilon _0}{\gamma _{\text{e}}}{q^3}/2\Omega _{\text{B}}^2$ with *q* $ \simeq $ 2*k*_1_ and ${\Omega _{\text{B}}}$ being the Brillouin frequency shift of the material. Also, $\alpha = \delta + i\beta $ is the complex damping coefficient where $\delta \left( \Omega \right) = \Omega {\Gamma _{\text{B}}}q/2\Omega _{\text{B}}^2$ and $\beta \left( \Omega \right) = \left( {\Omega _{\text{B}}^2 - {\Omega ^2}} \right)q/2\Omega _{\text{B}}^2$ with ${\Gamma _{\text{B}}}$ representing the Brillouin linewidth of the material and $\Omega = {\omega _2} - {\omega _1} &lt; 0$. The common solution for equation (5) assumes that the envelopes of the optical and acoustic fields do not vary significantly over the mean acoustic propagation distance $2\pi /\delta \left( {\Omega = {\Omega _{\text{B}}}} \right)$, so that $\Delta \rho $ is the spatial steady-state solution which is

\begin{equation*}\frac{{i\eta {A_1}A_2^*}}{{\delta + i\beta }}.\end{equation*}

This assumption is justified for long acousto-optic interaction lengths (depth of field of several micrometers). By inserting equation (6) into the differential equation of *A_2_* in equation (5), *A*_2_(z) can be obtained and the SBG spectrum |*A*_2_(*z*= 0)/*A*_2_(*z* = *L*)|^2^ is then expressed as

\begin{equation*}{{\text{e}}^{2\kappa \eta \frac{\delta }{{{\delta ^2} + {\beta ^2}}}{{\left| {{A_1}} \right|}^2}L}},\end{equation*}

where *L* is the acousto-optic interaction length. By approximating the exponential function with the first-order Taylor series expansion, the small-signal SBG spectrum $G\left( \Omega \right)$ is readily given by

\begin{equation*}2\kappa \eta \frac{\delta }{{{\delta ^2} + {\beta ^2}}}{\left| {{A_1}} \right|^2}L.\end{equation*}

Substituting the definitions of $\kappa $, $\eta $, $\delta $, $\beta $ into equations (7), (8) and using a Lorentzian function to approximate the spectral shape of the SBG line $ - $ i.e. the spectral factor $\delta \left( \Omega \right)/\left( {\delta {{\left( \Omega \right)}^2} + \beta {{\left( \Omega \right)}^2}} \right) - $we obtain an expression for $G\left( \Omega \right)$ that is consistent with the literature [3], i.e.

\begin{equation*}{g_0}\frac{{{{\left( {{\Gamma _{\text{B}}}/2} \right)}^2}}}{{{{\left( {{\Omega _{\text{B}}} - \Omega } \right)}^2} + {{\left( {{\Gamma _{\text{B}}}/2} \right)}^2}}}{I_1}L,\end{equation*}

where the SBG line-center factor *g*_0_ is $\gamma _{\text{e}}^2\omega _2^2/nv{c^3}{\rho _0}{\Gamma _{\text{B}}}$, and the pump intensity *I*_1_ is $2n{\varepsilon _0}c{\left| {{A_1}} \right|^2}$. We note that *g*_0_ and hence the peak SBG ${G_{\text{B}}} = G\left( {{\Omega _{\text{B}}}} \right)$ are closely related to the mean mass density, ${\rho _0}$. Hereafter, $G\left( \Omega \right)/{I_1}L$ is referred as the SBG line factor $g\left( \Omega \right)$ with units of m W^−1^.

### 2.2. Signal-to-noise ratio (SNR)

We define the SNR for cw and pulsed SBS microscopy as the ratio of the squared peak SBG signal to the noise variance added to the SBG lineshape. Assuming that the noise variance is dominated by the shot noise of the Stokes beam intensity, the SNR is

\begin{equation*}\frac{{\mu {{\left( {{g_0}f \times \mathop \int \limits_{ - \infty }^\infty {I_1}\left( t \right){I_2}\left( t \right){\text{d}}t \times L} \right)}^2}{\tau _D}}}{{f\mathop \int \limits_{ - \infty }^\infty {I_2}\left( t \right){\text{d}}t}},\end{equation*}

with $\mu $ representing the detection coefficient with units of m^2^/joule, *f* the repetition rate of the pump and Stokes beams, *I*_1_(*t*) and *I*_2_(*t*) the temporal profile of the pump and Stokes intensities with duration ${\boldsymbol{\tau}}$ much larger than the phonon lifetime (<∼1 ns in aqueous samples at 780 nm), and ${\tau _{\text{D}}}$ is the detection time of a spectral point. For cw-SBS microscopy, the SNR in equation (10) reduces to [10]

\begin{equation*}\mu G_{\text{B}}^2\langle {I_2}\rangle {\tau _{\text{D}}},\end{equation*}

where the angular brackets denote time averaging, and the peak SBG is ${G_{\text{B}}} = {g_0}\langle {I_1}\rangle L$. For pulsed-SBS microscopy with a square temporal profile of duration $\tau $, the SNR is given by equation (11) with the peak SBG ${G_{\text{B}}} = {g_0} \times \langle {I_1}\rangle /f\tau \times L$, where $f \times \tau $ is the duty cycle ($0 &lt; D \unicode{x2A7D} 1$) of the pump and Stokes intensities, and $\left\langle {{I_1}} \right\rangle /f\tau $ is the peak pump intensity, $I_1^{{\text{peak}}}$ [34, 35]. We note that long-pulse excitation increases the peak SBG compared to cw excitation by *D* and thus the SNR by *D* ^2^. This increase is a result of SBS interaction of the peak, rather than the average, pump and Stokes intensities with the acoustic wave. As a result, long-pulse excitation can achieve similar SNR to cw excitation but with lower average power, reducing linear phototoxicity over time.

**Figure 1.**
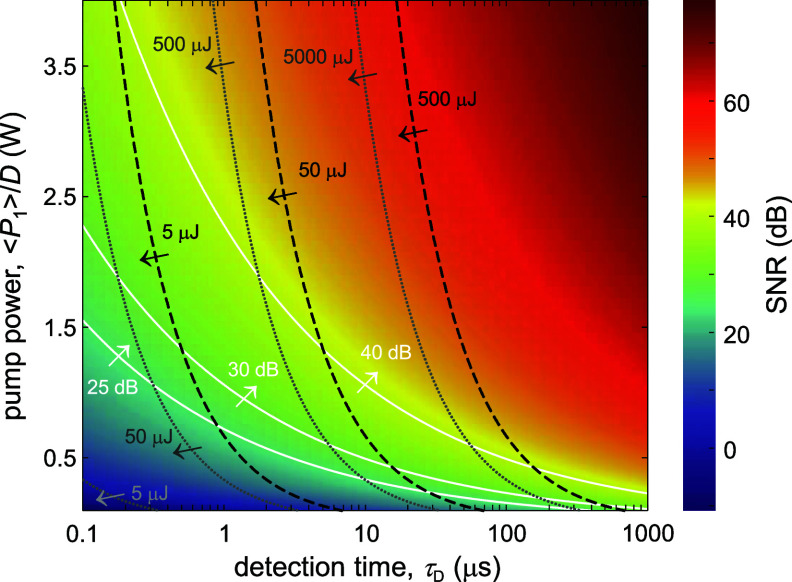
SNR in SBS microscopy as a function of pump power and detection time. The pump power represents the average power for duty cycle *D* = 1, and the peak power otherwise. White solid lines, contours of SNR. Values increase with arrow direction. Black dash lines, contours of total excitation energy under long-pulse excitation, assuming duty cycle *D* of 5%. Gray solid lines, contours of total excitation energy under cw excitation. Values decrease with arrow direction. The average pump and Stokes powers are at a ratio of 2:1 from the overall excitation power to maximize the SNR. The SNR was calculated using equation (11) with reference to an SNR of 35 dB measured in water with average pump and Stokes powers of 250 mW and 15 mW at ${\tau _{\text{D}}} = 100 \ \mu s$.

Figure 1 shows the dependence of SNR on pump power and spectral integration time, where *D* = 1 corresponds to cw excitation. For example, using cw excitation or long-pulse excitation with *D* = 5% of 0.25 W pump power, an SNR of ∼30 dB can be obtained for ${\tau _{\text{D}}}$ = 100 $\mu $s, resulting in an overall excitation energy of about 5000 $\mu $J or under 500 $\mu $J, respectively for cw and long-pulsed excitation. This represents more than one order of magnitude energy reduction under long-pulse excitation, as expected from a duty cycle lower than 10%.

### 2.3. Precision of the Brillouin shift and linewidth, and mean mass density

In this subsection, we derive lower bounds on the achievable precision of the Brillouin shift and linewidth, as well as the mean mass density values as obtained from the measured SBG spectrum following least squares fitting. To this end, the gradient equations for the three parameters $\left( {{\theta _1},{\theta _2},{\theta _3}} \right) = \left( {{{\hat \Omega }_{\text{B}}},{{\hat \Gamma }_{\text{B}}},{{\hat \rho }_0}} \right)$ were expressed and set to zero, with the model $S\left( {{\omega _i};{\theta _1},{\theta _2},{\theta _3}} \right)$ for the data set $\left( {{\Omega _i},{s_i}} \right)_{i = 1}^N$ expanded to first order in each parameter alone around the true value of the parameters $\left( {\theta _1^0,\theta _2^0,\theta _3^0} \right) = \left( {{\Omega _{\text{B}}},{\Gamma _{\text{B}}},{\rho _0}} \right)$. Here, ${\Omega _i}$ is the *i*th sample of the angular frequency $\Omega $ and ${s_i}$ is the corresponding SBG measured. By isolating $\Delta {\theta _k} = {\theta _k} - \theta _k^0,$ $k = 1,2,3$ from these equations, we obtain

\begin{equation*}\Delta {\theta _k} = \frac{{\sum\nolimits_{i = 1}^N {{r_i}{{S^{\prime}}_{{\theta _k},i}}} }}{{\sum\nolimits_{i = 1}^N {{{S^{\prime}}_{{\theta _k},i}}^2} }},\end{equation*}

where the residual ${r_i} = {s_i} - S\left( {{\omega _i};\theta _1^0,\theta _2^0,\theta _3^0} \right)$ and $S_{{\theta _k},i}^{\prime}$ is the partial derivative of the model with respect to ${\theta _k}$ evaluated at $\left( {\theta _1^0,\theta _2^0,\theta _3^0} \right)$. Squaring equation (12) and calculating the expectation value under the assumption that the residuals are normally, identically, and independently distributed with zero mean and variance ${\sigma ^2}$, the precision of $\Delta {\theta _k}$ can be proven to be

\begin{equation*}\frac{\sigma }{{\sqrt {\sum\nolimits_{i = 1}^N {{{S^{\prime}}_{{\theta _k},i}}^2} } }}.\end{equation*}

Noteworthy, equation (13) is consistent with that obtained in the localization analysis of point emitters [36].

**Figure 2.**
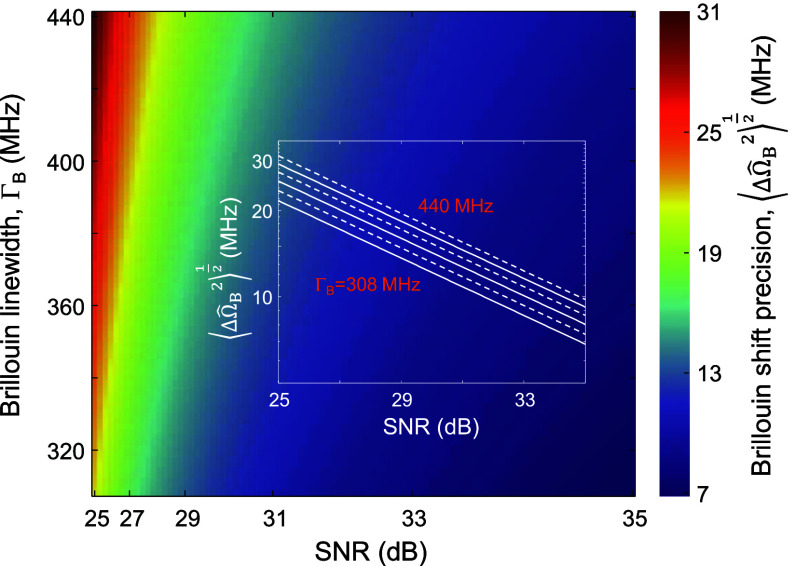
Brillouin shift precision in SBS microscopy as a function of the spectral width and the SNR of the SBG spectrum. The inset shows the dependence of the Brillouin shift precision on the SNR for different Brillouin linewidths in a log-log scale. The slope of the lines is ½.

We used equation (13) to evaluate the lower bounds on the precision of the Brillouin shift and linewidth, and mean mass density following least squares fitting. Figure 2 presents the Brillouin shift precision $\sqrt {\langle {\Delta \hat \Omega _{\text{B}}^2} \rangle }$ against the Brillouin linewidth ${\Gamma _{\text{B}}}$ and the SNR. We employed typical values for the SNR and for the linewidth of agar/water gels (0%–10% of agar) [17]. As biological materials typically contain a high fraction of water (>75%), the calculated precision represents an approximated lower bound for biological matter. The calculation used the Lorentzian model [equation (9)] in equation (13). As expected, $\sqrt {\langle{\Delta \hat \Omega _{\text{B}}^2}\rangle}$ improves with decreasing ${\Gamma _{\text{B}}}$ and increasing SNR and follows a $1/\sqrt {{\text{SNR}}} $ law, as observed in the inset of the figure.

**Figure 3.**
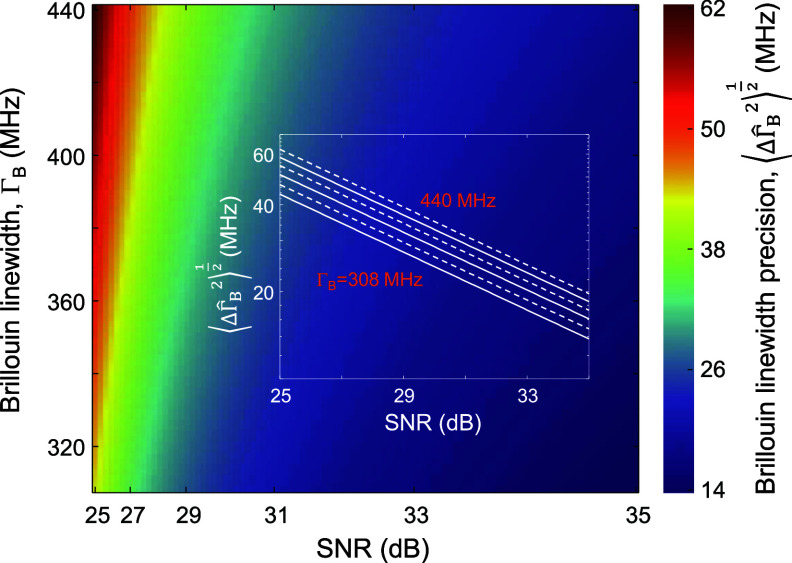
Brillouin linewidth precision in SBS microscopy as a function of the spectral width and the SNR of the SBG spectrum. The inset shows the dependence of the Brillouin linewidth precision on the SNR for different linewidths in a log-log scale. The slope of the lines is ½.

The dependence of the Brillouin linewidth precision $\sqrt {\langle {\Delta \hat \Gamma _{\text{B}}^2} \rangle}$ on the Brillouin linewidth ${\Gamma _{\text{B}}}$ and the SNR is shown in figure 3.

As before, a Lorentzian lineshape was used for modeling the SBG spectrum. $\sqrt {\langle{\Delta \hat \Gamma _{\text{B}}^2} \rangle}$ presents a similar behavior to $\sqrt{\langle {\Delta \hat \Omega _{\text{B}}^2}\rangle} $ (figure 2), but at approximately twice larger precision values due to the nearly fourfold smaller sum squared sensitivity of the Lorentzian model for the linewidth parameter [i.e.the denominator of equation (13)].

**Figure 4.**
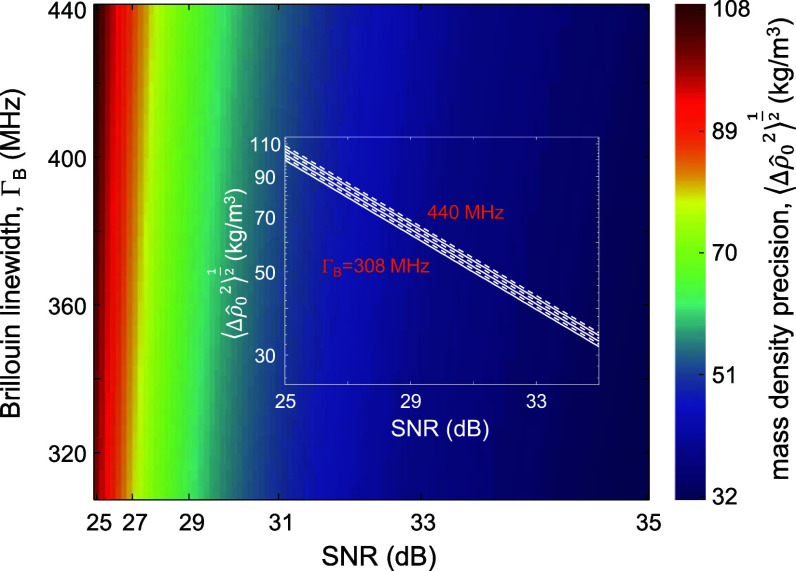
Mean mass density precision in SBS microscopy as a function of the spectral width and the SNR of the SBG spectrum. The inset shows the dependence of the mean mass density precision on the SNR for different linewidths in a log-log scale. The slope of the lines is ½.

As described in [17], it is possible to extract the mean mass density from the peak SBG, *G*_B_ , measurement assuming that the electrostrictive constant $\gamma $_e_ is estimated through use of the Lorentz–Lorenz law and that the refractive index of the medium is known. Figure 4 depicts the lower bound for the precision of the estimate of the mean mass density $\sqrt {\langle{\Delta \hat \rho _{\text{0}}^2}\rangle} $ against the Brillouin linewidth ${\Gamma _{\text{B}}}$ and the SNR. Also here, we used a Lorentzian lineshape to model the SBG spectrum. $\sqrt {\langle{\Delta \hat \rho _{\text{0}}^2}\rangle} $ exhibits the same rate law of $1/\sqrt {{\text{SNR}}} $ as $\sqrt {\langle{\Delta \hat \Omega _{\text{B}}^2}\rangle} $ and $\sqrt {\langle{\Delta \hat \Gamma _{\text{B}}^2}\rangle} $ at a fixed Brillouin linewidth but shows a lower increasing rate with linewidth at a constant SNR.

### 2.4. NA-induced spectral effects

**Figure 5.**
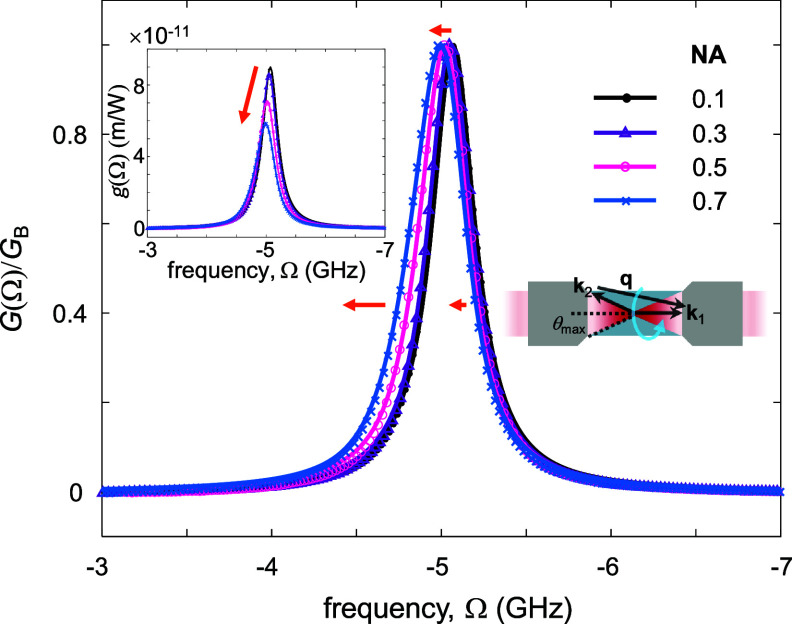
Measured (symbols) and calculated (solid lines) small-signal SBG spectra of water at different NA values. SBG values are normalized to the peak SBG *G*_B_ in the main panel. The SBG line factor $g\left( \Omega \right)$ is depicted in the left inset. The spread in the acoustic wavevectors is shown in the right inset. Orange arrows indicate the direction and strength of the NA effect on the SBG line. The Brillouin shift, linewidth, and peak SBG line factor extracted from the SBG spectra presented are (5.0650 GHz, 318 MHz, 8.92 × 10^−11^ m W^−1^) at NA = 0.1, (5.0467, 325, 8.59 × 10^−11^ m W^−1^) at NA = 0.3, (5.0175 GHz, 370 MHz, 7.05 × 10^−11^ m W^−1^) at NA = 0.5, (4.9865, 392 MHz, 5.88 × 10^−11^ m W^−1^) at NA = 0.7. Reproduced from [37]. CC BY 4.0.

In SBS microscopy, the focusing of the pump and Stokes beams to a finite spot size affects the shape of the SBG spectrum. Three spectral effects are induced (i) a shift of the spectrum towards low frequencies (in absolute values), (ii) an asymmetric broadening of the SBG line with a skewed tail towards low frequencies (in absolute values), and (iii) a reduction of the peak SBG [37]. These effects become more significant as the NA of the microscope objective lenses increases (i.e. larger ${\theta _{{\text{max}}}}$ in the right inset of figure 5) due to the increase of the spread in the acoustic wave vectors. Figure 5 shows measured and calculated small-signal SBG spectra $G\left( \Omega \right)$ of water at different NA values. The calculation of $G\left( \Omega \right)$ involves its description as the inhomogeneous superposition of spectral lines with Brillouin shift, linewidth, and peak gain that depend on the acoustic wavenumber |**q**| = |**k**_1_ $ - $ **k**_2_| and whose strength depends on the Fourier components of the pump and Stokes fields and on the point-spread function size [37]. In the main panel, we observe the increasing shift and asymmetric spectral broadening of $G\left( \Omega \right)$ (normalized to its peak SBG) towards the low frequency region (in absolute values) with increasing NA. The left panel describes the decrease in the measured and calculated peak gain of the SBG line factor $g\left( \Omega \right)$ with increasing NA.

In general, the observed SBG spectrum is the convolution of the Brillouin line, the pump and Stokes spectral profiles, and a scanning frequency function with width of ${\text{d}}\Omega /{\text{d}}t \times {\tau _{\text{D}}}$. The latter contribution to the spectral broadening of the intrinsic Brillouin line is up to several tens of MHz [17, 35], whereas that of the pump and Stokes spectral profiles is less than a few MHz for cw-SBS microscopy [17] and a few tens of MHz for pulsed-SBS microscopy [35]. Using an NA of 0.7, the NA-induced contribution to the spectral broadening of the intrinsic Brillouin line is ∼75 MHz, resulting in a total spectral broadening of ∼100–150 MHz [17, 35]. We note that these values define the effective spectral resolution of the SBS microscope, which compare favorably with those of 90°/forward scattering schemes [38], although the practical spectral resolution in aqueous samples is smaller due to the width of the liquid’s intrinsic Brillouin line, which is ∼300 MHz at 780 nm for water.

By approximating the Brillouin line as a Lorentizan with a full-width at half-maximum of 300 MHz$ - $which is generally acceptable in liquid-like materials [1, 39]–and the spectral transfer function of the microscope as a Lorentizan with full-width at half-maximum matching the total spectral broadening, the practical spectral resolving power of SBS microscopy in specimens with a high water content is ∼200–225 MHz at 780 nm. The latter approximation is applicable for the NA of the microscope used (0.7) and the cw and long pulse excitations, as supported by the agreement between the high adjusted coefficient of determination (0.998, 1) and the low square root of residual sum of squares (0.25, 0.21) for the Lorentzian and the aperture-broadened fits to the measured Brillouin line of water at NA = 0.7 [37].

## 3. Modalities of SBS microscopy

In this section, we describe two experimental realizations of SBS microscopy using either cw or pulsed pump and Stokes beams. Characterization of the precision of the Brillouin shift and/or linewidth is also presented. We note that current implementations of SBS microscopy employ a backscattering geometry (i.e. counterpropagating pump and Stokes waves) with identically circularly polarized pump and Stokes waves defined in a single reference frame (or opposite circular polarizations along the opposite directions of propagation of the pump and Stokes waves) to maximize the SBG and to support the propagation of longitudinally polarized acoustic waves, depending on spatial confinement effects [40]. In addition, 780 nm is used as penetration depth is higher and phototoxicity is lower than at visible wavelengths. Finally, current SBS microscopes enable three-dimensional imaging by raster-scanning the optical focus, e.g. via a mechanical stage onto which the sample is mounted.

### 3.1. cw SBS microscopy

**Figure 6.**
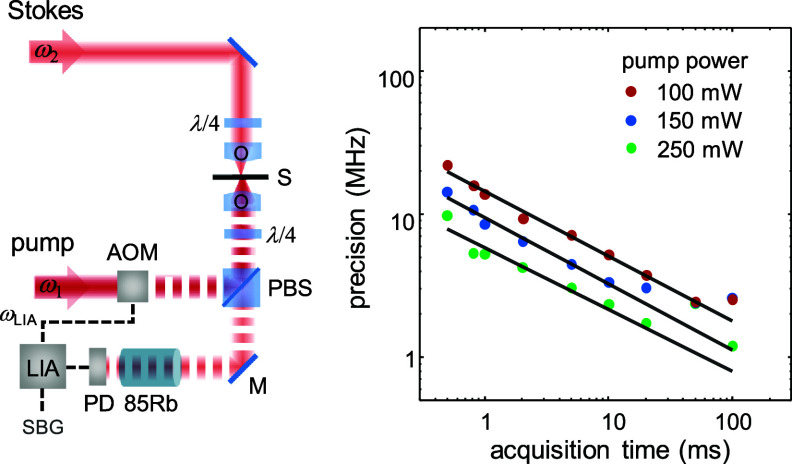
cw-SBS microscopy. Left, schematic of the setup. The pump laser at ${\omega _1}$ is frequency locked to a rubidium absorption line, and the Stokes laser is frequency scanned around ${\omega _2}$, where ${\omega _2} - {\omega _1}$ is near a Brillouin resonance. $\lambda $/4, quarter waveplate; O, microscope objective lens; PBS, polarizing beam splitter; AOM, acousto-optic modulator; M, mirror; 85Rb, rubidium 85 cell; PD, photodetector; LIA, lock-in amplifier at frequency ${\omega _{{\text{LIA}}}}$. Right, Experimental Brillouin shift precision at constant probe power (∼20 mW) with varying pump power and acquisition times obtained from 100 SBG spectra of water. Black solid lines represent fit of the data, confirming shot noise limited performances of the system down to millisecond exposures. Reproduced from [13]. CC BY 4.0.

The first implementations of SBS microscopy have used cw lasers, as illustrated in the left panel of figure 6 [14, 17]. SBS microscopes of this kind consist of two cw, single-frequency lasers, generally at 780 nm where laser sources are commercially available with narrow linewidth and >30 GHz tunability range which are co-focused into a sample in a counterpropagating geometry. The pump beam has a fixed frequency while its amplitude is modulated by an acousto-optic modulator. The Stokes frequency instead is scanned around the resonance of an acoustic wave of the material under test. The transmitted probe beam, detected by an amplified photodetector, contains a cw component and an SBS-mediated signal modulated at the frequency of the modulator. As a result, the SBG signal can be extracted from it using a lock-in amplifier referenced to the pump modulation frequency, and the Brillouin spectrum is recorded through scanning of the probe frequency. Usually, additional techniques are necessary to reduce parasitic background, e.g. from modulated pump stray light and photothermal process, which is also present in stimulated Raman scattering (SRS) and results in background that is extraneous to the nonresonant background in coherent anti-Stokes Raman scattering. For this purpose, atomic gas cells are very effective as they can be used to frequency lock the pump laser and thus are a natural solution for high-throughput and narrow-band notch filters at the pump frequency [13]. For subcellular imaging, the pump and Stokes beams are focused into the sample using high NA objective lenses (0.7), resulting in diffraction-limited optical resolution (∼ 0.3 × 0.3 × 2 *μ*m^3^).

As described in sections 2.1 and 2.2, cw-SBS microscopes operate in the weak nonlinear regime, and the small-signal SBG signal is proportional to the product of pump and probe powers [equation (8)]. Nevertheless, cw-SBS microscopes reach shot-noise-limited behavior down to millisecond exposures, as shown in the right panel of figure 6. This may not represent a large improvement over state-of-the-art spontaneous Brillouin microscopes, but they come with superb ability to also measure linewidth and gain at high precision [17], enabling additional contrast mechanisms for all-optical mechanical imaging [41]. To go beyond shot-noise limit, Li *et al* [42] recently demonstrated a quantum-enhanced version of SBS, which uses two quantum-correlated beams of light produced via four-wave-mixing in atomic vapors; one of such beams is used as a probe beam to be overlapped with a counterpropagating laser beam within a sample as in classical SBS; the other ‘conjugate’ beam serves as a reference for detection. Thanks to the intensity-difference squeezing between probe and conjugate beam a 3.4 dB SNR improvement was achieved compared to the non-squeezed case, although absolute SNR and performance was not superior to classical SBS implementations.

### 3.2. Pulsed-SBS microscopy

**Figure 7.**
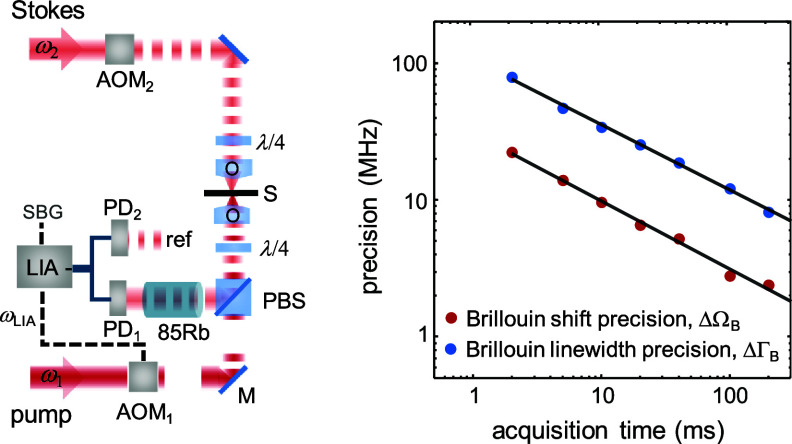
Pulsed-SBS microscopy. Left, setup schematic. The photodetector outputs are connected to a LIA via differential inputs, realizing a balanced detection. AOMs are synchronized by the same clock signal. ‘ref’ is a beam tapped from the pulsed Stokes laser. Right, measured Brillouin shift/linewidth precisions against acquisition time of water SBG spectra. Precision is calculated as the standard deviation of the Brillouin shift/linewidth determined from the Lorentzian fits of 300 SBG spectra measured sequentially. Pulse width and repetition rate, 60 ns and 1.1 MHz. Average pump and Stokes powers, 20 mW and 7 mW. The slope of the regression lines (black solid lines) is ∼0.5, confirming shot noise limited detection. Reproduced from [35]. CC BY 4.0.

As outlined in section 2.2, introducing a pulsed pump-probe approach allows to significantly increase the SNR compared to cw-SBS at equivalent average overall laser powers. Specifically, pulsed-SBS increases the peak SBG compared to cw excitation by the duty cycle $D = f \times \tau $, where *f* and $\tau $ are the repetition rate and duration of the pump and Stokes pulses, and consequently, the SNR by *D* ^2^. As a result, quasi-pulsed excitation could produce the same SBG as cw excitation but using high peak power and low average power, reducing linear photodamage. Nevertheless, the narrow linewidth of the Brillouin interaction (hundreds of MHz) requires nanosecond pulses rather than picosecond or femtosecond lasers used in other nonlinear microscopies. Thus, available improvements in SBG are orders of magnitude lower than in other optical nonlinear effects. Pulsed-SBS has recently been experimentally realized by ‘quasi-pulsing’ both the pump and Stokes cw-lasers, either by utilizing acousto-optic modulators and lock-in detection [35] or electro-optic modulators (Pockels cells) and boxcar averaging [34], achieving duty cycle *D* of 4%–5% at a repetition rate *f* of ∼1 MHz. A schematic setup of pulsed-SBS microscopy is shown in the left panel of figure 7.

Apart from the introduction of quasi-pulsing, a second important element in pulsed SBS is a balanced detector which can substantially reduce relative intensity noise, yet at the expense of an increase of the rms (shot) noise by $\sqrt 2 $. Experimentally, pulsed-SBS is able to achieve an SNR of 32 dB with an overall excitation power of 18 mW (13 mW pump, 5 mW probe) at 20 ms acquisition time of the SBG spectrum, effectively 22-fold reduction in illumination powers compared to cw-SBS at shot-noise limited performance and otherwise similar effective spectral resolution and imaging speed [35]. In particular, spectral shift and linewidth precision on the order of ∼8 and 30 MHz, respectively for 20 ms acquisition time and 27 mW overall power can be obtained [34, 35] (see right panel of figure 7, compare figure 6). When combined with high NA (0.7) objectives, pulsed-SBS has been shown to achieve a mechanical spatial resolution of ∼0.5 *µ*m and ∼2.5 *µ*m in the transverse and axial direction, respectively, close to the optical diffraction-limit of ∼0.3 × 0.3 × 2 *μ*m^3^ [35], and similar to cw-SBS microscopy with the same NA. After accounting for the finite linewidth of the measured medium, the spectral resolution of pulsed-SBS was determined as ∼150 MHz, with dominant contributions from the broadening due to the NA of the objective lenses used (0.7) and the time constant of the lock-in amplifier.

## 4. Measurements and analysis

In this section, we present mechanical-contrast imaging in live, biological systems by SBS microscopy. Also, spectrum analysis methods used in SBS microscopy are described.

### 4.1. Mechanical-contrast imaging in living systems

**Figure 8.**
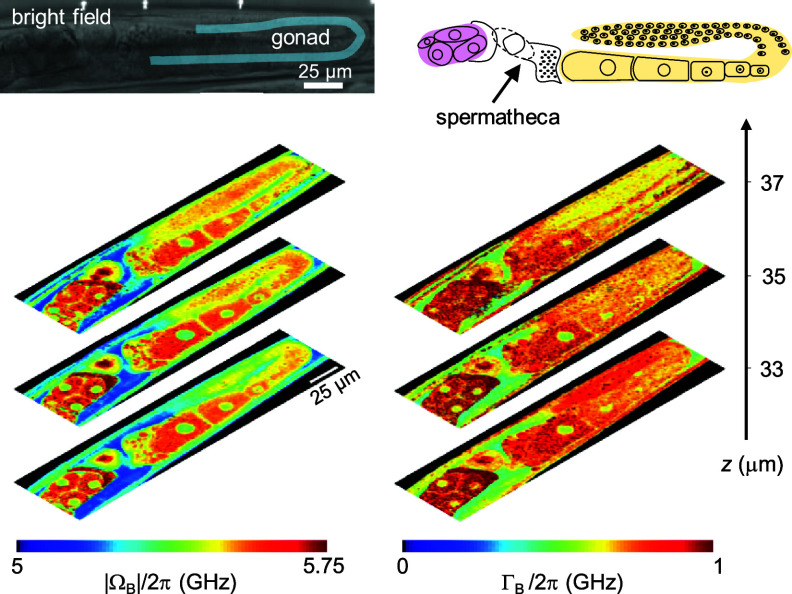
Cw-SBS microscopy data showing depth-resolved, subcellular images of the Brillouin shift (left) and linewidth (right) of the gonad arm of a live *C. elegans* nematode. Pixel-dwell-time is 20 ms. Total excitation power on the sample is ∼265 mW. Reproduced from [17], with permission from Springer Nature.

Since the first realization of a dedicated high spatial resolution and high-specificity SBS microscope for life science applications [17], SBS based bio-imaging has been demonstrated in a number of biological systems. cw-SBS microscopy has been successful in acquiring three-dimensionally resolved Brillouin maps of semitransparent living samples of up to tens of micrometers thickness with 20-ms pixel dwell times. Specifically, *Caenorhabditis elegans* worms were imaged at both the subcellular and organism scale with practical total recording times, showing the worm’s reproductive system (gonad, spermatheca, uterus, oocytes, and embryos) with excellent mechanical contrast and resolution, as observed in figure 8. Although sample absorption and scattering limit the strength of the SBS signal, tissue phantoms with attenuation coefficient of 45 cm^−1^ were also measured by SBS at SNRs of ∼15–25-dB and with 0.1–1-s spectrum acquisition times [11].

More recently, cw-SBS microscopy has been utilized to image individual cells with high Brillouin shift, linewidth, and peak gain contrasts, including NIH/3T3 fibroblasts [43], where a physics-driven model selection framework minimized spectral artifacts and facilitated the quantification and demixing of Brillouin spectra of different mechanical origin in the cells. Furthermore, by exciting acoustic phonons selectively, a mechanical contrast at the excitation frequency has been acquired in the sample with a significantly faster pixel dwell time (0.1 ms) and reduced excitation energy than cw and pulsed SBS microscopy. This selective excitation scheme has equally been shown to be well applicable to both living *C. elegans* nematodes as well as NIH/3T3 cells [41], where different tissues in the nematode’s head and subcellular features could be resolved.

**Figure 9.**
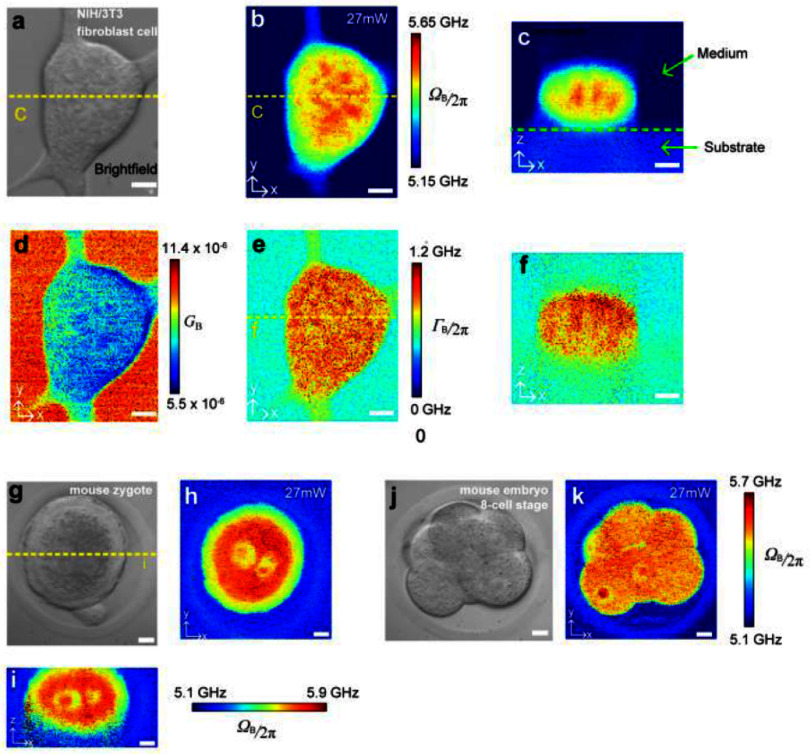
Pulsed-SBS microscopy data demonstrating imaging of fragile biological samples. (a)–(f) Images of cultured NIH/3T3 fibroblast cells, showing (a) brightfield, Brillouin shift (b), (c) and linewidth (e), (f) of a transversal and axial cross-section, respectively. (d) Shows the Brillouin gain. Scale bars in (a)–(f) are 5 *µ*m. (g)–(i) Pulsed-SBS imaging of mouse embryos, showing (g) brightfield and (h), (i) Brillouin shift of a transversal, and axial cross-section, respectively. (j), (k) Brightfield and Brillouin shift images of an 8-cell-stage mouse embryo. Scale bars in (g)–(k) are 10 *µ*m. Pixel-dwell-time is 20 ms. Total excitation power on the sample is ∼27 mW. Reproduced from [35]. CC BY 4.0.

At the same time, pulsed-SBS microscopy has been shown to be well suited to image Brillouin spectral maps at similar spatiotemporal resolution to cw-SBS microscopy but, due to the employed quasi-pulsing and resulting SNR enhancement, at substantially lower average optical illumination powers, which has further helped to enable applications to sensitive biological specimens. Specifically, the over 10-fold lower power levels employed have shown to be instrumental for dense spatial 3D and/or prolonged time-lapse imaging with 20-ms pixel dwell time, where total light exposure of the living specimens needs to be in general minimized to avoid thermal or phototoxic effects. Pulsed-SBS microscopy has generated high-resolution 3D Brillouin spectral maps of NIH/3T3 mouse fibroblasts [34, 35], primary human brain microvascular endothelial cells and mouse embryonic stem cells [35], as presented in figure 9.

Inside the cells, subcellular compartments with subtle mechanical differences such as the nucleus or nucleoli could be discerned. Furthermore, pulsed-SBS microscopy has enabled visualization of the mechanical properties of adult *C. elegans* and pre-implantation mouse embryos, as well as entire mouse mammary gland organoids and *C. elegans* embryos over 3 h during development [35].

Heterogeneous living tissues encompass different mechanical constituents, which require adequate, high spectral resolution to be distinguished. Here, pulsed-SBS microscopy has been able to reveal several tissue types inside the living zebrafish larvae notochord, including a highly rigid extra-cellular matrix, as well as the surrounding muscle segments including the spinal cord canal [35]. A further advantage of the pulsed-SBS scheme and its lower illumination power is that image artifacts due to optical trapping of dust, endogenous particles or even entire samples in the illumination laser light focus can be substantially decreased.

### 4.2. Analysis methods

In general, SBS microscopy produces an SBG spectrum for each spatial pixel. The extraction of the Brillouin shift, linewidth, and peak gain of the SBG line is the basis for the analysis of the SBS dataset. The algorithms used to analyze the SBS data stack can be classified to single or multiple SBG peak detection and estimation. As these algorithms are based on the least-squares fitting method, high SNR and high practical spectral resolution are required, as presented in sections 2.2–2.4. Single SBG peak estimation with least-squares fitting has been directly used on SBG spectra of liquids, cells, and organisms acquired using medium to high NA objective lenses to retrieve pixelwise the spectral parameters of the SBG line, i.e., the Brillouin shift ${\Omega _{\text{B}}}$, linewidth ${\Gamma _{\text{B}}}$, and peak gain ${G_{\text{B}}}$, with adequate precisions of ∼10 MHz, ∼20 MHz, and ∼9 $ \times $ 10^−7^ [43]. Typically, a Lorentzian lineshape is used as the model function in the fitting procedure, which is acceptable in liquid-like materials with low and medium NA acquisitions [37], as also observed in table 1 that summarizes the values of ${\Omega _{\text{B}}}$, ${\Gamma _{\text{B}}}$, and ${G_{\text{B}}}$ measured in water by a cw-SBS microscope and extracted using different model functions for the SBG spectrum acquired.

**Table 1.** Mean $ \pm $ std of ${\Omega _{\text{B}}}$, ${\Gamma _{\text{B}}}$, ${G_{\text{B}}}$ of water using 99 SBG spectra measured by a cw-SBS microscope with 0.7-NA objectives [43]. Different model functions are applied in the least squares fitting, resulting in SNRs of ∼28–30 dB.

	${\Omega _{\text{B}}}$ (GHz)	${\Gamma _{\text{B}}}$ (MHz)	${G_{\text{B}}}$
Lorentzian	5.027 $ \pm $ 0.0077	367 $ \pm $ 17	2.73 $ \times $ 10^−5^ $ \pm $ 8.25 $ \times $ 10^−7^
Damped harmonic oscillator	5.037 $ \pm $ 0.0078	365 $ \pm $ 17	9.98 $ \times $ 10^−6^ $ \pm $ 4.13 $ \times $ 10^−7^
Aperture broadened	5.042 $ \pm $ 0.0081	387 $ \pm $ 18	2.67 $ \times $ 10^−5^ $ \pm $ 8.21 $ \times $ 10^−7^

When multiple SBG peaks are present in a single spectrum (e.g. at interfaces between mechanically different materials), the standard, single lineshape fit to the SBG spectrum, results in an inaccurate estimate for the Brillouin parameters. In particular, the estimated Brillouin shift is then a weighted sum of the individual Brillouin shifts of the mechanically different materials in the scattering volume of the sample and its surroundings. Consequently, washout in the contrast of material boundaries occurs.

To address this problem, the simultaneous estimation of multiple SBG peaks using least-squares fitting has recently been proposed. Here, a peak detection step prior to estimation of the spectral parameters of the multiple peaks is required. The detection step determines the number of peaks in the SBG spectrum measured. One peak detection algorithm analyzes the zero crossings of the first spectral derivative of the fitted sum of Lorentzian functions to decide on the number of extrema in the measured spectrum [35]. A different peak detection algorithm uses model selection between single and multiple peak lineshapes based on the Akaike information criterion with a statistical threshold derived from the SBG line of water to faithfully approximate the spectrum acquired [43]. An important point in the least-squares fitting of multiple peaks is the frequency range over which the composite SBG spectrum is recorded. Frequency ranges of 2–4 GHz are typically used [17, 34, 35, 43], with larger ranges generally improving the estimation of the spectral parameters of the multiple SBG lines, likely because of the inclusion of the heavy tails of the model function in the fitting procedure.

There are a few difficulties associated with the simultaneous fitting of multiple SBG peaks: (i) the more peaks one likes to simultaneously fit, the larger the number of parameters, making least-squares optimization more computationally intensive, (ii) the accuracy and precision of the parameter estimates are prone to decrease with the increasing number of densely located peaks, and (iii) the error probability, i.e. the probability to erroneously detect the number of peaks in the SBG spectrum, increases with the increasing number of peaks [43]. To tackle the latter two points, the SNR should be increased.

## 5. Discussion

Here, we discuss experimental factors that determine the spatial, temporal, and spectral resolution of SBS microscopy. A brief comparison with spontaneous Brillouin, iSBS, and SRS microscopies is also presented.

The optical spatial resolution of SBS microscopy is determined by the excitation wavelength, the NA of the two objective lenses used to focus the pump and Stokes beams in the sample, and the nonlinearity of the SBG signal in the excitation intensity. The resulting optical spatial resolution is therefore equivalent to that of confocal spontaneous Brillouin microscopy using similar NA and excitation wavelength, where micrometer to submicrometer 3D optical resolution has been demonstrated [17, 20, 35]. In iSBS microscopy, the optical resolution is significantly reduced ($ \unicode{x2A7E} 10 \ \mu {\text{m}}$ [15, 16]) due to the large wavelength of the phonon generated. It is worth pointing out that the optical resolution and the mechanical resolution are in general not the same [29, 40].

The temporal resolution of SBS microscopy $ - $ defined as the pixel-dwell time or the acquisition time of an SBG spectrum in a single spatial pixel—is determined by the SNR [equation (11)] required for faithful fitting of the Brillouin spectrum, and the frequency range over which it is measured. For SNR of ∼30 dB and a frequency range of 2–4 GHz, a temporal resolution of 20 ms has been achieved in biological settings [17, 34, 35]. A comparable temporal resolution has been accomplished also by confocal spontaneous Brillouin microscopy using VIPAs and electron-multiplying charge-coupled device cameras [44]. Recently, at SNRs of ∼30 dB, SBS microscopy with selective excitation of acoustic phonons has been demonstrated with a temporal resolution of 0.1 ms in living samples [41]. In iSBS microscopy with cw probing, the temporal resolution is determined by the SNR, the repetition rate of the excitation light pulse, and the response time of the photodetector and the oscilloscope. For precise measurements of the Brillouin shift, a temporal resolution of ∼3 ms has been shown to be feasible in hydrogels [45]. We note that the frequency range in iSBS microscopy does not affect temporal resolution and is controlled by the temporal width of the excitation light pulse.

As described in section 2.4, assuming good SNR spectra (>25 dB), the actual spectral resolution of SBS microscopy is determined by the intrinsic width of the measured Brillouin lines at the excitation wavelength, the NA-induced spectral broadening, the pump and Stokes spectral profiles, the scanning rate of the pump-Stokes difference frequency, and the detection time of a spectral point of the SBG spectrum. An actual spectral resolution of ∼200–225 MHz has been estimated in high water content samples at 780 nm using NA of 0.7 [17, 35]. In confocal spontaneous Brillouin microscopy based on Fabry Perot interferometers, the practical spectral resolution depends on the intrinsic linewidth of the recorded Brillouin spectra, the NA-induced spectral broadening, and the excitation laser line and spectrometer profiles. In addition to the former parameters, the pixel spectral dispersion also affects spectral resolution in VIPA-based Brillouin microscopes, leading to an actual spectral resolution of ∼275 MHz in water at 780 nm in best conditions (low NA of 0.3 and single VIPA) [31]. In iSBS microscopy, the practical spectral resolution is determined by the intrinsic width of the acquired Brillouin lines at the excitation wavelength, the NA-induced spectral broadening which is maximum for forward scattering, and the observable time range controlled by the repetition rate of the excitation light pulse. The latter contribution is less than 10 MHz for an observable time range larger than 100 ns.

Despite the similarities between SBS and SRS processes, SBS differs from SRS in (i) the energy of the phonon involved, (ii) the characteristics of vibrational modes, (iii) the gain bandwidth, and (iv) the scattering geometry. These differences determine the features of SBS and SRS microscopies. For example, the preferable imaging mode in SBS microscopy is transmissive (owing to the phase matching requirement and the shorter phonon wavelength), whereas SRS microscopy operates in either transmissive or epi modes. Also, the detection of scattered phonons is more complicated in SBS microscopy than in SRS microscopy because of the large difference in energy between optical and acoustic phonons. Finally, due to the long damping time of SBS (nanoseconds) relative to that of SRS (picoseconds), SBS microscopy uses quasi-cw excitation (i.e., long nanosecond optical pulses) for obtaining adequate gain levels, whereas SRS microscopy employs picosecond light pulses, enabling high image acquisition speeds at biocompatible excitation powers.

## 6. Conclusions and future perspectives

Coherent Brillouin light scattering based microscopy methods presented in this article enable new features which are promising for contactless, cross-sectional, label-free, all-optical mechanical measurements and imaging of materials and biological systems with high sensitivity, specificity, and imaging speed. These features include (i) detection of the coherent Brillouin signals against a near-dark background, providing high contrast for weak signals, (ii) high effective spectral resolution as no spectrometer is involved, enabling high mechanical specificity, e.g. at interfaces or mixtures of mechanically different materials, (iii) long pulse and/or selective excitation of acoustic phonons, significantly reducing the average excitation energy, and hence potential linear photodamage, as well as being attractive for video rate measurements and imaging.

To further advance coherent Brillouin microscopy, some challenges need to be addressed. First, despite recent improvements, the imaging speed of SBS microscopy still falls short of other microscopy methods, and additional speed-ups require further improvements in the SNR. A way to tackle this is by developing dedicated single-frequency, yet rapidly tunable, high peak power, pulsed lasers at wavelength and repetition rate suitable for SBS imaging. Second, SBS microscopy systems are relatively complicated in terms of design and data acquisition and analysis. Simpler pump and probe lasers and field programmable gate array and/or graphics processing unit based processing and analysis can streamline the experimental setups and provide real-time analysis. Furthermore, machine learning can facilitate and improve on standard analysis methods currently employed for SBS microscopy, although extensive training data sets are often required in the first place. Third, in addition to instrument development, inclusion of suitable microscope incubators, particularly for longitudinal studies, and optimization of protocols for imaging and measurements of material/biological samples are needed, including the integration of SBS microscopy with other measurement and imaging methods, such as phase and fluorescence imaging and flow cytometry. Fourth, to make selective excitation of acoustic phonons a powerful tool for biological research and discovery, mapping of different mechanical components and dynamic mechanical changes in biological specimens is necessary. This can be achieved by using prior knowledge on sample composition and spectral Brillouin measurements of mechanical constituents and dynamic mechanical changes in calibrated samples. Finally, we expect that the similarities between SBS and SRS will foster new developments in SBS microscopy along the lines of SRS microscopy.

## Data Availability

The data that support the findings of this study are available upon reasonable request from the authors.
